# What it means to abandon race in science?

**DOI:** 10.1113/EP091489

**Published:** 2024-05-03

**Authors:** Michael Yudell, Evelynn M. Hammonds

**Affiliations:** ^1^ College of Health Solutions Arizona State University Phoenix Arizona USA; ^2^ Department of the History of Science Harvard University Cambridge Massachusetts USA

A February 2024 paper in the journal *Nature*—‘Genomic data in the All of Us Research Program’— has come under fire for a figure seen by some as reinforcing biological views of human races (Kozlov, 2024). The figure, titled ‘Genetic ancestry in All of Us’, uses six racial groups—Asian, Black or African American, Middle Eastern or North African, Native Hawaiian or Other Pacific Islander, White, and More than one population—to represent, by race, the genetic diversity of the All of Us participant population (All of Us Research Program Genomics Investigators, 2024).

We will not debate the accuracy of the figure here, or its role in reinforcing biologized notions of race considered by many anathema to the field. Nor is it worth relitigating the ongoing misuse of racial categories in scientific thought or how such categories create hierarchy where there is none, reinforcing crude notions of difference that very poorly reflect human genetic diversity in general or the relationship between that diversity and health‐related traits (Vyas *et al.*,^2020^; Yudell et al., 2016).

Instead, the figure stands as an example of how the use of race persists despite more than a century of scientific evidence illustrating its limitations and dangers (Graves, 2001; Hammonds & Herzig, 2009; Yudell, 2014). And it persists despite a 2023 U.S. National Academies of Sciences, Engineering, and Medicine (NASEM) report, *Using Population Descriptors in Genetics and Genomics Research: A New Framework for an Evolving Field* (National Academies, 2023), which was supported by the National Institutes of Health in the United States, the institute that created and financially supports the All of Us Project.

We do appreciate that the National Academies report and its recommendations are barely a year old. Science is a lumbering field. Science policy perhaps more so. Our hope, and the hope of others who had a hand in calling for and/or shaping the report, is that its recommendations would, over time, help reshape how geneticists operationalized population descriptors in their research. That a centerpiece of human genetic disease research at the NIH largely ignored the report's recommendations speaks to the ongoing challenges of redressing these failures in the field.

The report itself tries to settle a longstanding challenge in scientific and health research: should racial terminology—terms like White, Black, Asian and Hispanic—be used to describe and measure human biological diversity in genetics and genomics research? (Borrell *et al.*, 2021). The answer, according to the report itself, is a resounding no. In almost all cases, scientists *should not* be using race in genetics and genomics research (National Academies, 2023, p. 102).

Why is this?

Well, for two reasons. First, as the National Academies Committee explains, race has been shown to be a flawed proxy for one's genetic make‐up and biology. ‘There is a pervasive misconception that humans can be grouped into discrete, innate biological categories’, the report states. This is because, ‘the fundamentally sociopolitical origins of these categories make them a poor fit for capturing human biological diversity and as analytical tools in human genomics research’ (National Academies, 2023, p. 7). And, second, the use of racial descriptors in genetics research has the dangerous side effect of promoting the mistaken belief that races reflect fundamental differences, both biological and social, between human groups. The report seeks to put such notions to rest, stating that the ‘use of these categories reinforces misconceptions about differences caused by social inequities. Current practices in human genetics, including the use of descriptors such as continental ancestry, also reinforce these views’ (National Academies, 2023, p. 7).

While the *Nature* paper cited above suggests that progress is slow, the NASEM report lays out 12 recommendations for how to improve the study of human genetic diversity and *not* reinforce biologized racial concepts. The first set of recommendations (nos 1–5) focus on the limits of typological thinking in genetics and are among the report's most important. Typological thinking assumes that every individual is a perfect example of a specific type and this can represent a group. Recommendation 1 could not be clearer in rejecting race, stating plainly that ‘researchers should not use race as a proxy for human genetic variation’. The recommendation sends a message to researchers that race *should not* be used as a proxy and that researchers *should not* use such categories ‘whether self‐identified or not’. Recommendations 2 and 3 build on this, telling researchers that ‘when grouping people in studies of human genetic variation, researchers should avoid typological thinking’ and that they ‘should be attentive to the connotations and impacts of the terminology they use to label groups’. Recommendation 4 focuses on the relationship between genetic information and other health determinants calling upon ‘researchers conducting human genetics studies should directly evaluate the environmental factors or exposures that are of potential relevance to their studies, rather than rely on population descriptors as proxies’. The recommendation also calls for more collaborative and community‐based research, calling on genetic and genomic researchers to collaborate with ‘experts in the social sciences, epidemiology, environmental sciences, or other relevant disciplines to aid in these studies, whenever possible’ (National Academies, 2023, pp. 101–112).

The second set of recommendations (6–8) focus on the selection and use of population identifiers in research, specifically on tailoring their use ‘to the type and purpose of the study’, on applying them ‘consistently to all participants’, and disclosing ‘the process by which they selected and assigned group labels and the rationale for any grouping of samples’ (National Academies, 2023, pp. 113–116).

The final set of recommendations (9–13) focus on implementation and accountability, calling upon funders, institutions where research is conducted, and scientific journals to help facilitate implementation and to provide incentives for the types of interdisciplinary collaborations needed. NASEM also call on funders of science to ‘create new initiatives to advance the study and methods development of best practices for population descriptor usage in genetics and genomics research’ (National Academies, 2023, pp. 158–159).

For geneticists and those working in related fields, on page 121 of the report, there is a useful table designed to support researchers as they make decisions about how and when to use different population descriptors, including race (National Academies, 2023, pp. 121). For those considering how to balance population level research with concerns about how to study health disparities that impact racial and ethnic groups differentially, the table acknowledges that race can be used as a population descriptor. But as the report makes abundantly clear, research should adhere to strict criteria when utilizing racial categories. For example, the committee concludes that ‘racial or ethnic phenotypic differences cannot be ascribed to genetic differences without evidence’, and that scientists should ‘avoid racial or ethnic labels because they are poor proxies for differences in environmental exposures’ and instead ‘replace or supplement descent‐associated population descriptors with information about the relevant factors that mediate differences in environmental exposures, such as education, types of employment, housing quality, and access to health care, to name only a few’. However, the committee acknowledges that ‘when the goal is explicitly to study the effect of structural racism and discrimination, then racial and ethnic labels may be appropriate but need to be carefully described (e.g., self‐identified or not) and justified’ (National Academies, 2023, pp. 130–131). These criteria can be a guidepost across an array of scientific, clinical and public health related fields regardless of whether genetics is a component of the research being done.

Whether the report is ultimately successful in promoting this much‐needed change in genetics and genomics research, however, will depend on several factors. First, the success of the report's recommendations will depend on the willingness of the National Institutes of Health and other federal funders of science to support the transition away from racial typologies in scientific study. This will require the NIH to use its budgetary and policy authority to create substantive and sustained change in how human populations are studied, described and recruited across an array of disciplines. Second, scientists must be willing partners in this space, following the guidelines for the use of race and population descriptors in the NASEM report, in reports and recommendations for other professional groups, and from a burgeoning literature in natural and social scientific fields involved in the study of human populations. Third and finally, there must be a commitment in the genetic and genomics community to collaborate with partners in public health and social sciences to integrate methods from these fields into the study of health disparities so we can truly move past racial typologies in science.

As public health scientists and scholars whose work centers on the history and ethics of public health, we are both delighted and relieved that such significant changes have the imprimatur of the National Academies, and optimistic that the details of this report can help improve the scientific study of human diversity. Abandoning biological race concepts in scientific study will take time and dedication. And through efforts like that of the NASEM, these necessary changes can and will happen.

## AUTHOR CONTRIBUTIONS

Both authors have read and approved the final version of this manuscript and agree to be accountable for all aspects of the work in ensuring that questions related to the accuracy or integrity of any part of the work are appropriately investigated and resolved. All persons designated as authors qualify for authorship, and all those who qualify for authorship are listed.

## CONFLICT OF INTEREST

The authors declare they have no conflicts of interest.

## FUNDING INFORMATION

None.
